# Hospital nursing factors associated with decreased odds of mortality in older adult medicare surgical patients with depression

**DOI:** 10.1186/s12877-022-03348-1

**Published:** 2022-08-13

**Authors:** Aparna Kumar, Douglas Sloane, Linda Aiken, Matthew McHugh

**Affiliations:** 1 Thomas Jefferson University College of Nursing, 901 Walnut Street St. Suite 800, Philadelphia, PA 19107 USA; 2 Center for Health Outcomes and Policy Research, University of Pennsylvania School of Nursing, 418 Curie Blvd., 2L, Philadelphia, PA 19104 USA

**Keywords:** Nursing, Older adult, Mortality, Depression

## Abstract

**Background:**

Depression is common, costly, and has deleterious effects in older adult surgical patients. Little research exists examining older adult surgical patient outcomes and depression and the potential for nursing factors to affect these outcomes. The purpose of this study was to determine the relationship between hospital nursing resources, 30-day mortality; and the impact of depression on this relationship.

**Methods:**

This was a retrospective cohort study employing a national nurse survey, hospital data, and Medicare claims data from 2006–2007. The sample included: 296,561 older adult patients, aged 65–90, who had general, orthopedic, or vascular surgery in acute care general hospitals from 2006–2007, 533 hospitals and 24,837 nurses. Random effects models were used to analyze the association between depression, hospital nursing resources, and mortality.

**Results:**

Every added patient per nurse was associated with a 4% increase in the risk-adjusted odds of mortality in patients with depression (*p* < 0.05). Among all patients, every 10% increase in the proportion of bachelor’s prepared nurses was associated with a 4% decrease in the odds of mortality (*p* < 0.001) and a one standard deviation increase in the work environment was associated with a 5% decrease in the odds of mortality (*p* < 0.05).

**Conclusions:**

For older adult patients hospitalized for surgery, the risk of mortality is associated with higher patient to nurse ratio, lower proportion of BSN prepared nurses in the hospital, and worse hospital work environment. Addressing the mental health care needs of older adults in the general care hospital setting is critical to ensuring positive outcomes after surgery. Hospital protocols to lower the risk of surgical mortality in older adults with and without depression could include improving nurse resources.

## Background

Among older adults hospitalized for surgery, depression is common, affecting nearly one in four older adults [1, 2]. Surgery increases the likelihood of morbidity and mortality in older adults, more so than in younger adults [1]. In addition, nearly 50 million surgical procedures annually will result in adverse events; orthopedic, general, and vascular surgical procedures are especially fraught with risk [3–5]. Depression increases these risks in older adults and is associated with longer length of stay [6, 7], increased risk of readmission [8], adverse events [9, 10], higher healthcare spending, and increased hospitalizations [11, 12]. These risk factors make nursing care of the older adult surgical patient especially crucial.

The Registered Nurse (RN) is central to the care of these vulnerable patients and the detection of potential complications. It is hypothesized that RN surveillance, defined as synthesizing complex patient data to help shape clinical decisions, is the mechanism through which this is achieved [13, 14]. RNs carefully monitor patients in the postoperative period and can help lower the risk of complications, death, and failure to rescue (FTR). Availability of organizational resources that support good nursing care, including a good work environment, adequate staffing, and a high proportion of bachelor’s prepared nurses (BSNs), can enable RNs to more effectively perform surveillance and thereby lower the odds of complications and death, especially in vulnerable older adults with depression [15–19]. A large body of research has demonstrated that the organization of nursing resources (work environment, proportion of BSNs, and good staffing) are associated with better surgical patient outcomes and fewer adverse events [16, 18]. Staffing in particular has been associated with lower risk of mortality among general, orthopedic and vascular surgical patients, both among patients with and without mental illness [16, 20].

Better staffing levels are conceptualized to improve the ability of the nurse to monitor patients or perform *surveillance* [21]*.* Nurse surveillance is particularly critical for patients with depression. Depression exacerbates the risks of delirium, delayed wound healing, anesthesia complications, increased pain perception, and increased risk of adverse events [4, 22–24]. Patients with depression may also have lower levels of social support and decreased desire to perform activities of daily living (ADLs), which can decrease engagement in recovery and rehabilitation [8, 25]. In older adult patients, this is further complicated by atypical clinical presentation of depression, including irritability, anxiety, and somatic complaints as opposed to depressed mood [25]. If not recognized immediately after surgery, patients with depression have a still greater risk of delirium, complications, and physical health deterioration [25]. Thus, the role of the nurse in delivering timely and sensitive care to older adult surgical patients with depression, is critical.

The purpose of this retrospective cohort study was to determine the relationships between hospital nursing resources (work environment, proportion of BSNs, and good staffing) and mortality in older adult surgical patients with and without depression. We hypothesized that better organization of hospital nursing would be associated with lower risk of mortality, and that the relationship between one or more of these resources and mortality would be more pronounced for patients with depression than for patients without depression.

## Methods

### Design and Sample

This study employed a retrospective cohort design linking three data sets: 1) the 2006–2007 Multi-State Nursing Care and Patient Safety Study Survey; 2) Medicare hospital claims data from 2006–2007; and 3) the 2006–2007 American Hospital Association (AHA) Annual Survey. The AHA annual survey is a survey sent to nearly 6,200 hospitals and 400 health systems and gathers information on utilization, organizational structure, setting, and facilities [26]. The patient sample included 296,561 Medicare beneficiaries who underwent surgery in 533 acute care hospitals in four states (California, New Jersey, Pennsylvania, and Florida) [27]. This methodology has been previously established with linked data sets and samples of nursing respondents from the Multi-State Nursing Survey [16].

### Patients

Medicare fee-for-service beneficiaries (traditional Medicare recipients) with Medicare as the primary payer, between the ages of 65 and 90 hospitalized for common surgical procedures (orthopedic, general, and vascular) from 2006 and 2007 in California (CA), New Jersey (NJ), Pennsylvania (PA), and Florida (FL) were included in the analysis if they did not leave against medical advice [27–29]. Patients received surgery at the hospital where payment was submitted for reimbursement (Inpatient Prospective Payment System) based on the procedure that they received [30]. Index admissions were identified for 296,561 patients who were admitted for orthopedic, general, or vascular surgery. If patients had multiple admissions, one was randomly chosen. To ensure there were no readmissions, there could be no other admissions in the previous 30 days. Choosing the commonly received surgical groupings allows for comparison across hospitals where the surgeries were performed as well as offers established models for risk adjustment [28].

### Hospitals

533 Acute care hospitals from CA (*n* = 193), NJ (*n* = 69), PA (*n* = 133), and FL (*n* = 138) were included if they: 1) had at least 100 surgical discharges from September 2005 to November 2007; 2) had data on the AHA Survey and the Medicare data set and 3) had more than 10 nurse respondents [16, 19, 31]. Nurse responses to the 2006–2007 Multi-State Nursing Care and Patient Safety Study Survey were from 24,837 nurses – an average of 47 per hospital—working in direct patient care in the study hospitals and ensured sample heterogeneity [16, 31]. Responses were aggregated and analyzed at the hospital level [27]. The sample was considered representative as over 70% of hospitals in each state were included, and were where the vast majority of surgical procedures occurred in those states. Hospitals not represented were small hospitals with few nurse respondents [16].

### Measures

#### Outcome variables

Mortality (30-day all-cause mortality) was defined as all deaths within 30 days of admission, in or outside the hospital [27, 28].

#### Explanatory variables

Nurse education was measured by the proportion of nurses in each hospital who reported having at least a BSN. The nurse staffing measure represented the average of each nurses’ report of the number of assigned patients on the unit on their last shift divided by the number of nurses on the unit for the same shift, aggregated to the hospital level [19]. The nurse work environment was measured by the Practice Environment Scale of the Nursing Work Index (PES-NWI), a 31-item measure endorsed by the National Quality Form, which has demonstrated reliability and predictive validity [16, 18, 32, 33]. It includes five subscales representing features of the work environment: nurse participation in hospital affairs, nursing foundations for quality of care, nurse manager ability, leadership and support, staffing and resource adequacy, and nurse physician relations [32]. Responses from individual nurses were aggregated to the hospital level and a mean score was created for each subscale as well as the composite scale at the hospital level.

### Risk adjustment

Both hospital and patient characteristics were included as control variables in analytical models for risk adjustment. Hospital characteristics included: number of hospital beds, teaching status, and technology status [27]. Patient characteristics included: age, sex, and 26 of 27 Elixhauser comorbidities (excluding depression in this analysis) [34].

### Depression (Moderator)

The depression indicator was drawn from the Medicare Chronic Condition Warehouse (CCW) flag, which is derived from complete patient claim file records (not just hospital claims) from 2006–2007, for patients who received a diagnosis of depression within the calendar year prior to the identified index admission [27]. The CCW flags cover a variety of common conditions and are derived from Medicare and Medicaid Services (CMS) administrative claims data. The depression variable was included if a patient received a diagnosis of depression prior to the index admission (or CCW flag). The absence or presence of depression was indicated with a dichotomous variable, coded 0 and 1 respectively. By employing the CCW flag, Medicare data from both the inpatient and outpatient encounters were included in the data set, increasing the proportion of patients with depression identified compared to hospital claims alone (15% vs. 7%).

### Analysis

Descriptive statistics were calculated for hospitals and patients. Unadjusted and adjusted random effects models were employed to examine the relationships between hospital nursing resources (work environment, staffing, and education individually and jointly) and 30-day mortality among depressed and non-depressed adult surgical patients, accounting for other hospital and patient characteristics [27]. We estimated the main effects of the three different nursing resources, and used interaction terms to evaluate whether any of the three resource measures had different effects for depressed and non-depressed patients. Model (1) is unadjusted, and estimates these differences without controlling for the other hospital characteristics and patient features listed above and in the note below the table. Models (2), (3) and (4) all adjust for other hospital and patient characteristics, the latter of which consist of over 90 factors including age, sex, race, transfer status, and indicator variables for procedure types [(Diagnosis Related Groups (DRGs)], and Elixhauser comorbidities. While Model (1) is a “main effects” model which excludes any interactions, models (3) and (4) include different forms of the interaction that was found to be significant, between staffing and depression. Model (2) allows the interaction to be unconstrained, or for the effect of staffing to differ for depressed and non-depressed patients. The interaction in Model (3), by contrast, from which the insignificant main effect of staffing is dropped – and having coded the non-depressed patients 0 and the depressed patients 1 – constrains the interaction and specifies that the effect of staffing is present for depressed patients but not for non-depressed patients. Since all effects of our primary variables are significant in Model (3) or involved in a significant interaction, it is that model which is preferred to describe the effects that are present. We found that one of the three interaction terms was significant, involving the effect of staffing for depressed and non-depressed patients, and further found that the interaction involved an effect of staffing that was present for depressed patients but not for non-depressed patients [27]. We describe the results of the “constrained” interaction in the result section below. All analyses were done using STATA 15/IC, and significance was set at *p* < 0.05. The study was exempt from Institutional Review Board (IRB) review.

## Results

Of the 296,561 patients in the sample (Table 1), 14.6% (42,815) had a diagnosis of depression prior to surgery. The majority of patients were white (88.5%) and the mean age was approximately 77 years, both for patients with and without depression. A greater proportion of patients were female among patients with depression (72.1%) versus without depression (56.6%). The most frequent surgical procedures in the sample were hip operation excluding replacement (8.7%), major intestinal procedure (5.3%), and lower extremity and humerous procedure (5.2%). Many more patients with depression (14.0%) had hip operations compared to those without depression (7.8%). Mortality in the non-depressed (4.1%) and depressed (4.2%) groups of patients was comparable (*p* = 0.327).

**Table 1:** Surgical patient characteristics for non-Depressed (*n* = 253,746) and Depressed Patients (*n* = 42,815)

	All Patients n(%) *n* = 296,561	Non-Depressed n (%) *n* = 253,746	Depressed n (%) *n* = 42,815	*p* value
Age (years), mean(SD)	76.7 (6.6)	76.6 (6.6)	77.1 (6.8)	< 0.001
Sex				
Male	121,871 (41.1%)	109,922 (43.3%)	11,949 (27.9%)	< 0.001
Female	174,690 (58.9%)	143,824 (56.6%)	30,866 (72.1%)	< 0.001
Race				
White	262,376 (88.5%)	223,769 (88.2%)	38,607 (90.2%)	< 0.001
Black	15,442 (5.2%)	13,700 (5.4%)	1,742 (4.1%)	< 0.001
Other	18,743 (6.3%)	16,277 (6.4%)	2,466 (5.8%)	< 0.001
Transfer to/from Outside Hospital	1,181 (0.4%)	934 (0.4%)	247 (0.6%)	< 0.001
Major Surgical Category				
Orthopedic Surgery (MDC 8)	124,607 (42.0%)	103,519 (40.8%)	21,088 (49.3%)	< 0.001
General Surgery (MDC 6, 7, 9, 10)	144,513 (48.7%)	126,062 (49.7%)	18,451 (43.1%)	< 0.001
Vascular Surgery (MDC 5)	27,441 (9.3%)	24,165 (9.3%)	3,276 (7.7%)	< 0.001
Mortality	12,148 (4.1%)	10,357 (4.1%)	1,791 (4.2%)	0.327
Top 10 Procedures				
Major Vessel Operation Except Heart (MDC 5)	13,397 (4.5%)	12,679 (4.9%)	1,258 (2.9%)	< 0.001
Major Intestinal Procedures (MDC 6)	15,735 (5.3%)	13,826 (5.4%)	1,909 (4.4%)	< 0.001
Hip Operations Except Replacement (MDC 8)	25,761 (8.7%)	19,757 (7.8%)	6,004 (14.0%)	< 0.001
Cardiac Valve and Other (MDC 8)	6,837 (2.3%)	5,452 (2.1%)	1,385 (3.2%)	< 0.001
Back and Neck Spinal Procedure (MDC 8)	7,660 (2.6%)	6,579 (2.6%)	1,081 (2.5%)	< 0.001
Lower Extremity and Humerous Procedure (MDC 7)	15,301 (5.2%)	13,387 (5.3%)	1,914 (4.4%)	0.052
Lower Extremity Except Foot (MDC 7)	6,935 (2.3%)	6,202 (2.4%)	733 (1.7%)	< 0.001
Local Excision and Removal of Int Fix except Hip or Femur w/o CC/MCC (MDC 8)	7,578 (2.6%)	6,285 (2.5%)	1,293 (3.0%)	< 0.001
Local Excision and Removal of Int Fix Hip and Femur w/o CC/MCC (MDC 8)	9,303 (3.1%)	8,104 (3.2%)	1,199 (2.8%)	< 0.001
Soft Tissue Procedures with MCC (MDC 8)	11,530 (3.9%)	10,367 (4.1%)	1,163 (2.7%)	< 0.001

*Notes: SD* Standard Deviation, Percentages rounded and may not total 100%; *CC* complications or comorbidities, *MCC* major complications or comorbidities [27]

Data Sources: The Office of Statewide Health Planning and Development (OSHPD) in CA, the Agency for Health Care Administration (AHCA) in FL, New Jersey Department of Health and Senior Services (NJDHSS), and the Pennsylvania Health Care Cost Containment Council (PHC4)

Among the 533 study hospitals (Table 2), nearly half had more than 250 beds (45.8%), roughly half were non-teaching hospitals (51.8%) and had high-technology status (53.1%), defined as hospitals that offered open heart surgery, organ transplantation, or both, and more than seven in ten were non-profit (71.4%). Among hospitals studied the mean staffing ratio was 5:1, and on average 40% of nurses had at least a BSN. The mean PES-NWI score, measuring quality of the nurse work environment, was slightly above average (*M* = 2.75, *SD* = 0.2) [27].

**Table 2:** Numbers and percentages of hospitals and their characteristics and nursing factors

Hospital Characteristic	n (%)
Size	
≤ 100 beds	59 (11.1%)
101–250 beds	230 (43.2%)
> 250 beds	244 (45.8%)
Teaching Status	
Non-Teaching	276 (51.8%)
Minor Teaching	214 (40.2%)
Major Teaching	43 (8.1%)
Technology Status	
High Technology	283 (53.1%)
Low Technology	250 (46.9%)
Location	
Division	218 (40.9%)
Metro	261 (48.9%)
Micro	43 (8.1%)
Rural	8 (1.5%)
Ownership	
Government	49 (9.3%)
Non-Profit	375 (71.4%)
For-Profit	101 (19.2%)
State	
California	193 (36.2%)
Florida	138 (25.9%)
New Jersey	69 (12.9%)
Pennsylvania	133 (24.9%)
Hospital Nursing Factors, mean (SD)	
PES-NWI, mean (SD)	2.75 (0.20)
Poor (*n* = 178)	2.49 (0.11)
Mixed (*n* = 178)	2.72 (0.05)
Best (*n* = 177)	2.96 (0.12)
Staffing, mean (SD)	5.4 (1.3)
Education (% BSN), mean (SD)	39.7 (13.5)

*Notes:* Practice Environment Scale of the Nurse Work Environment (PES-NWI); PES-NWI excludes Staffing and Resource Adequacy Subscale. Nurse staffing is measured as the ratio of patients to nurses. BSN = Bachelors of Science in Nursing; Education is reported as the proportion of nurses holding a BSN at the hospital level. Location is defined by Core Based Statistics Area (CBSA): Division =  > 2.5 million, Metro = Metropolitan, 50,000–2.5 million; Micro = Micropolitan, 10,000–50,000; Rural =  < 10,000. Percentages rounded and may not total 100%; Number totals may not equal 533 due to missing information from the American Hospital Association (AHA)

Data Source: American Hospital Association (AHA); Multi-State Nursing Care and Patient Safety Study Survey [27]

**Fig. 1:**
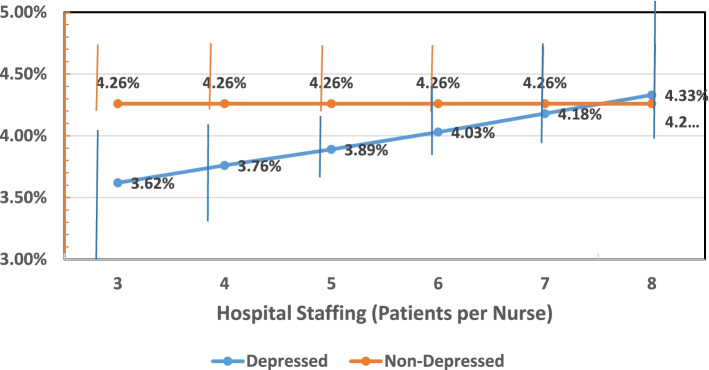
Estimated percentage of deaths at different staffing levels, for depressed and non-depressed patients

Table 3 displays the odds ratios from four different random effects models indicating the differences in the odds on mortality for depressed and non-depressed patients, and the differences in those odds for hospitalized patients with different hospital nurse work environment (PES-NWI), different percentages of BSN nurses, and different levels of hospital-level staffing. Model (3) indicates that there are significant associations between the work environment and mortality [OR = 0.95, CI = (0.91–0.99), *p* < 0.01] and the proportion of BSNs and mortality [OR = 0.96, CI = (0.93–0.98), *p* < 0.001]. Thus, each standard deviation increase in the hospital work environment score decreases the odds on mortality by 5%, and every 10 percentage point difference in BSN nurses score decreases the odds on mortality by 4%. While the main effect of depression is only marginally significant, and the main effect of staffing is nil, the significant interaction in the model implies that for depressed patients each additional patient per nurse in the hospitals in which they were cared for was associated with a 4% increase in their odds of mortality [OR = 1.04, CI = (0.1.01–1.09), *p* < 0.01]. This interaction also implies a significant difference between depressed and non-depressed patients in hospitals with better staffing (or lower nurse workloads) but not in hospitals with poorer staffing. This is depicted graphically in Fig. 1.

**Table 3:** Odds Ratios from random effects models indicating the effects of depression, the practice environment, nurse staffing and nurse education on the odds of 30-day mortality

	**Unadjusted Model**		**Adjusted Model (1)**		**Adjusted Model (2)**		**Adjusted Model (3)**	
	**OR(CI)**	**P**	**Adjusted for patient/hospital characteristics OR (CI)**	**P**	**Adjusted for patient/hospital characteristics with Unconstrained Interaction OR (CI)**	**P**	**Adjusted for patient/hospital characteristics with Constrained Interaction OR (CI)**	**P**
**Depression**	1.02 (0.97–1.08)	0.385	0.96 (0.90–1.01)	0.104	0.95 (0.90–1.00)	0.083	0.95 (0.90–1.00)	0.085
**PES-NWI**	0.92 (0.88–0.96)	< 0.001	0.95 (0.91–0.99)	0.008	0.95 (0.91–0.99)	0.008	0.95 (0.91–0.99)	0.01
**BSN10**	0.96 (0.94–0.99)	0.002	0.96 (0.93–0.98)	< 0.001	0.96 (0.93–0.98)	< 0.001	0.96 (0.93–0.98)	< 0.001
**Staffing**	0.99 (0.97–1.02)	0.608	1.00 (0.97–1.02)	0.753	0.99 (0.96–1.02)	0.420	^***^	
**Interaction Staffing x Depression**					1.05 (1.01–1.09)	0.034	1.04 (1.01–1.08)	0.047

*Notes:* Depression is indicated by the presence of a Chronic Condition Warehouse (CCW) depression flag. The PES-NWI is the Practice Environment Scale of the Nurse Work Index (excludes the Staffing and Resource Adequacy Subscale), measured in 1 standard deviation unit increments. Staffing is the ratio of patients to nurses and is a continuous measure. Education is the proportion of BSNs at the hospital level, measured in 10% increments. Patient characteristics include: age, sex, race, transfer status, procedure type (DRG), and Elixhauser comorbidities. Elixhauser comorbidities included: Congestive heart failure, cardiac arrhythmia, valvular disease, peripheral vascular disorders, pulmonary circulation disorders, hypertension complicated/uncomplicated, paralysis, other neurological disorders, chronic pulmonary disease, diabetes complicated/uncomplicated, hypothyroidism, renal failure, liver disease, peptic ulcer disease excluding bleeding, AIDS, lymphoma, metastatic cancer, solid tumor without metastasis, rheumatoid arthritis/collagen vascular disease, coagulopathy, obesity, weight loss, fluid and electrolyte disorders, blood loss anemia, deficiency anemias, alcohol abuse, drug abuse, psychoses (depression-excluded in this analysis). Hospital characteristics include: teaching status, technology status, size, location (CBSA), ownership, state, proportion of medical surgical and proportion of ICU nurses at the hospital level. Unadjusted models include the PES-NWI, staffing, and education only. Jointly adjusted models jointly adjust for the PES-NWI, staffing, and education and patient and hospital characteristics. Jointly adjusted models with the interaction jointly adjust for the PES-NWI, staffing, and education and patient and hospital characteristics in addition to the interaction between staffing and depression. OR: Odds Ratio; CI: Confidence Interval [27]

## Discussion

Our study demonstrated that better nurse staffing and nurse work environments as well as a higher proportion of hospital nurses with bachelor’s education qualifications were associated with decreased odds of mortality among older adults undergoing surgery, consistent with prior studies [16, 19, 35]. We additionally demonstrated that the effect of staffing in particular was pronounced among patients with depression, suggesting an increased vulnerability to poor surgical outcomes in this population and the potential for nursing care to have a greater impact among patients with depression.

The findings of this study are particularly salient. Depression, common among older adults, is poorly recognized and treated [1]. While greater focus has been placed on the screening of depression in primary care over the past decade [27, 36–38], this has been less apparent in the hospital setting. Yet, hospitals are intervention sites from which to leverage existing infrastructure and resources, namely nursing, to improve patient outcomes for patients with depression.

Nursing surveillance in the hospital is particularly important for older adult surgical patients, especially patients with depression whose physiological vulnerabilities are higher, further increasing the risk of infection and adverse events [4, 20, 21, 27, 39]. Adequate staffing may allow for such care. When nurses have fewer patients, they can provide more individual attention and may be more likely to focus on their psychosocial needs. When workloads are too high, research suggests that nurses are unable to consistently complete tasks such as “adequate surveillance” or “administer medications on time” for patients, specifically when they practice in less than ideal environments or care for more patients [40, 41]. Nurses also have the potential to screen for depression and promote self-care in individuals with depression [42, 43]. They can identify risks specific to patients with depression like delirium, which is exacerbated by exposure to anesthetic agents in surgery [4, 23]. At the policy level, this study further supports lower patient to nurse staffing ratios to improve quality of care and lower mortality [27, 31].

Beyond the larger influence of staffing on mortality, other factors that may influence outcomes among patients with depression also warrant further discussion. There are physiological vulnerabilities which predispose patients with depression to complications [27, 39, 44]. Yet, there may be proportionally fewer patients with depression among surgical patients, who are typically a healthier population [27, 45]. Additionally, it is conceivable that complications leading to mortality were not captured and disproportionately affect patients with depression [27]. For example, a greater percentage of patients with depression were admitted for hip fracture, which could likely be attributed to a fall, perhaps not captured by 30 day mortality [27, 46, 47]. Psychosis also happens more often among patients with depression, most often caused by delirium [45, 48]. While delirium is a medical condition and can be fatal, it is likely that delirium is detected in the hospital setting and resolved more quickly than other complications [23, 27]. Therefore, lower staffing ratios should be promoted, but so should nurse driven interventions to address physiological vulnerabilities, decrease bias and stigma, address delirium, and reduce mortality in this high-risk population [27]. Screening patients for depression prior to surgery could allow such nurse driven interventions to be implemented to prevent negative sequelae [49].

Certain limitations should be noted. This was a retrospective cohort analysis and causality cannot be determined [27]. Still, the combined data set employed demonstrated a novel approach to studying depression, which may be less likely to be coded due to clinical presentation, billing bias, or favoring higher reimbursement diagnoses [27, 50]. The CCW identifier for depression in the Medicare data set helped to identify more patients with both inpatient and outpatient data as well as a broader range of diagnostic codes than is traditionally included [27, 50]. One final limitation is the age of the study data, although it is unlikely that fundamental relationships have changed over time. The CCW flag is no longer operating as a methodology for utilizing the flags to identify chronic conditions. Data collection with CCW flags ceased in 2012. Therefore, the opportunity to utilize this flag, which identified administrative claims data for depression in both outpatient and inpatient settings was unique. A new nurse respondent survey was not available until 2016. Hence the nurse survey data could not have been linked as years did not match. In addition, this study examined the influence of nursing factors on outcomes for patients with depression. Hospital size, organization, technology status may change, however, these variables are controlled for in the final models. Furthermore, the influence of nurse staffing on patient outcomes has been well documented and is understood as influential across multiple contexts and in the international setting [35]. Finally, the model of studying organizational context and nurse respondents as a proxy for organizational quality has not changed over time. This model developed by Dr. Linda Aiken and colleagues has been supported by decades of research [24, 35], suggesting that the approach employed in the study is consist with previous approaches. While organizational features may change over time as a response to payer and provider pressures as well as the broader socio-political landscape, our models adjusted for organizational, patient, and hospital factors, thus leaving the study to examine the relationship of nursing at the organizational level to patient outcomes. We do not anticipate that patients with depression themselves change over time nor do the impacts of staffing on the care of patients.

To the authors’ knowledge, no prior study has examined the differential impact of nursing factors on mortality among patients with and without depression [20]. While the results of this study suggest that better staffing levels are associated with lower odds of mortality among patients with depression, the underlying factors that drive this relationship are not known [27]. In this study, it was theorized that the care, observation, and assessment of the nurse drives this relationship. However, this was not tested in this retrospective cohort study. Further avenues for research may examine missed care, or tasks that nurses are unable to complete due to understaffing, and their impact on outcomes for patients with and without depression. Such research may clarify the impact of staffing on task completion. Furthermore, knowing that patients with depression may have inherent physiological vulnerabilities, chart abstraction could provide additional clinical data on these vulnerabilities [27]. An example could be poor wound healing, common among patients with depression, which may not have been captured in the claims data. Such factors may contribute to mortality.

Surprisingly, in this study, there was not a significant effect of staffing on mortality among the non-depressed patients. This runs counter to the many studies which have found such effects [16–19, 31, 33]. There are a few considerations. First, many of the studies finding an effect in non-depressed patients use a broader age range of all adult patients instead of just older adults as in this study. In studies using a similar older adult population, significant effects have been found relative to mortality [51, 52] and there are other outcomes where staffing has been shown to be significant—readmissions and length of stay (LOS) are examples [51–53]. It is also possible that the staffing effect is conditional on other factors such as the work environment [16]. Thus not finding a significant effect shouldn’t be taken as staffing only matters for patients with depression.

## Conclusions

In sum, the results from this study demonstrate that each additional patient per nurse was associated with a 4% increase in the odds of death for older adult surgical patients with depression. Depression can increase the risk of complications following surgery for hospitalized older adults and increase the cost of care [27]. With increasing pressures for health systems to improve quality of care, especially among individuals with chronic illness such as depression, managing adverse events among patients with depression is important [27]. Depression can increase the complexity of care, worsen health outcomes, decrease daily function, and lower quality of life for older adult hospitalized patients, who may also have other complex comorbidities. At the organizational level, lowering patient to nurse ratios on medical floors where older adults with depression may be present, can help lower the risk of mortality in this population [27]. Equally important, this study supports training and equipping nurses with the skills to identify and integrate care for depression in older adult surgical patients with depression. This study supports evidence for health care administrators and policy makers to lower staffing levels and to continue to create interventions to improve outcomes in patients with medical illness and depression.

## Data Availability

The data that support the findings of this study are available from a combined data set from Centers for Medicare and Medicaid Services (CMS), the American Hospital Association (AHA), and the Center for Health Outcomes and Policy Research (CHOPR) at the University of Pennsylvania, School of Nursing but restrictions apply to the availability of these data, which were used under license for the current study, and so are not publicly available. Data are however available from the authors upon reasonable request and with permission of CMS, the AHA, and CHOPR.

## Abbreviations

ADL: Activities of Daily Living
AHA: American Hospital Association
BSN: Bachelor’s Prepared Nurses
CA: California
CCW: Chronic Condition Warehouse
CHOPR: Center for Health Outcomes and Policy Research
CMS: Medicare and Medicaid Services
DRG: Diagnosis Related Group
FL: Florida
FTR: Failure to Rescue
IRB: Institutional Review Board
LOS: Length of Stay
NIH: National Institutes of Health
NINR: National Institute of Nursing Research
NJ: New Jersey
PA: Pennsylvania
PES-NWI: Practice Environment Scale of the Nursing Work Index
RN: Registered Nurse
